# Efficacy and safety of Tibetan medicine Qingpeng ointment for acute gouty arthritis: protocol for a multi-center, randomized, double-blind, placebo-controlled trial

**DOI:** 10.1186/s13063-022-06338-1

**Published:** 2022-05-12

**Authors:** Ya-xi Shang, Xia Dong, Zhi-min Xie, Xiao-peng Li, Xin-chang Wang, Ji-yong Huang, Shu-feng Wei, Yuan Liu, Jian-ping Liu

**Affiliations:** 1 Centre for Evidence-Based Chinese Medicine, Beijing University of Chinese Medicine, 11 Bei San Huan Dong Lu, Chaoyang District, Beijing, 100029 China; 2 Fangshan Hospital, Beijing University of Chinese Medicine, Beijing, 102499 China; 3 The Second Affiliated Hospital of Zhejiang Chinese Medical University, Hangzhou, 310005 China; 4 Liuzhou People’s Hospital, Liuzhou, 545006 Guangxi Zhuang Autonomous Region China

**Keywords:** Gout, Acute gouty arthritis, Joint pain, Qingpeng ointment, Tibetan medicine, Randomized controlled trial

## Abstract

**Background:**

Acute gouty arthritis (AGA) is an inflammatory arthritis clinically characterized by severe pain, swelling, and restricted movement of joints, which may cause physical disability and decrease quality of life. The use of recommended first-line treatment agents for AGA may be limited by adverse events. There has been a traditional use of alternative therapies for AGA. Tibetan medicine Qingpeng ointment is one of the on-market herbal products used for symptom relief of AGA. Previous clinical studies indicated that Qingpeng ointment can relieve pain, swelling, redness, and dysfunction of joints in patients with AGA. However, there is no rigorous randomized trial to demonstrate its benefit for AGA. In order to evaluate the efficacy and safety of Qingpeng ointment for AGA, we designed a randomized controlled trial.

**Methods:**

This study is designed as a multi-center, randomized, double-blind, placebo-controlled trial. Two hundred and six adults with acute flare of gout, and visual analogue scale (VAS) score of joint pain ≥ 3 points will be recruited. Participants will be randomly assigned to herbal treatment or placebo group at a ratio of 1:1. Qingpeng ointment, or equal placebo ointment, will be applied topically at involved joints twice a day for consecutive 7 days. Patients in both groups would be allowed giving diclofenac sodium sustained-release tablets as rescue therapy when VAS score of joint pain ≥ 7 points during the treatment. The primary outcomes will be joint pain measured by VAS score, and joint swelling measured using width and thickness of affected joints and VAS score. Other outcome measures will be joint mobility, joint redness, C-reactive protein, serum uric acid, and the use of rescue medicine as well as adverse effect.

**Discussion:**

To the best of our knowledge, this study is the first multi-center, randomized, double-blind, and placebo-controlled clinical trial to assess the efficacy of Tibetan medicine Qingpeng ointment for AGA. The findings of this study would provide evidence for its use to relieve symptoms of AGA.

**Trial registration:**

ISRCTN ISRCTN34355813. Registered on 25 January 2021

**Supplementary Information:**

The online version contains supplementary material available at 10.1186/s13063-022-06338-1.

## Background

Gout is an inflammatory arthritis occurring in response to monosodium urate crystals formation. It has been recognized as the most common form of inflammatory arthritis [1], with a prevalence of 1.1 to 3.9% [2–6]. The disease burden of gout is rising globally [7]. Acute gouty arthritis (AGA) is clinically characterized by severe pain, redness and swelling of joints, and restricted movement, which may cause physical disability, as well as decreased quality of life [8]. Colchicine, nonsteroidal anti-inflammatory drugs (NSAIDs), and glucocorticoids are recommended as first-line treatment agents for AGA [9, 10]. However, comorbidities that may result in contraindications to these drugs are common in patients with gout [11]. Moreover, the use of these drugs may be limited by adverse events. For example, colchicine can increase the rate of diarrhea and gastrointestinal adverse events when compared with placebo and active comparators [12]. NSAIDs are associated with increased risk of gastrointestinal bleeding [13] and may exacerbate renal failure [14]. Glucocorticoids may cause weight gain, cardiovascular disease, diabetes, and infections [15]. Therefore, a complementary and alternative therapy that can effectively alleviate the symptoms of AGA with less side effects needs to be found.

Traditional Tibetan medicine (TTM) is a long-established system of traditional medicine with a history of more than 2000 years and is formed based on the beliefs and practices of the Tibetan culture [16]. TTM consists of numerous botanicals, animal medicines, and mineral medicines, in which many medicines are unique because of the unique growth environment [17]. TTM belongs to traditional minority medicine in China and plays an important role in the health care system in Tibet Autonomous Region and other Tibetan regions such as Qinghai and Sichuan Provinces in China [16]. Qingpeng ointment is a Tibetan patent medicine for external use, which is convenient and safe to use. Only a few patients who are allergic to the drug may experience skin irritation after using the drug. Qingpeng ointment consists of *Herba Oxytropis Falcatae* (Jidou), *Rhei Spiciforme Randix* (Yadahuang), *Radix Aconiti Flavi Et Penduli* (Tiebangchui), *Chebulae Fructus* (Hezi), *Terminaliae Belliricae Fructus* (Maohezi), *Phyllanthi Fructus* (Yuganzi), *Benzoinum* (Anxixiang), *Caulis Tinosporae* (Kuanjinteng), and artificial *Moschus* (Shexiang). Qingpeng ointment has the effects of promoting blood circulation, removing blood stasis, reducing swelling, and relieving pain and can be used to treat swelling and pain of joint and muscle caused by rheumatic arthritis, rheumatoid arthritis, osteoarthritis, and gout. Previous clinical studies indicates that Qingpeng ointment can relieve pain, swelling, redness, and dysfunction of joints in patients with AGA [18–23]; can relieve joint pain and swelling in patients with knee osteoarthritis [24, 25]; and can relieve joint swelling in patients with rheumatoid arthritis [26]. However, previous studies of Qingpeng ointment for AGA have some methodological deficiencies. In order to clearly evaluate the efficacy and safety of Qingpeng ointment for patients with AGA, it is necessary to design and conduct a randomized controlled trial with higher methodological quality.

## Objectives

The aim of this study is to design and conduct a double-blind, randomized, placebo-controlled trial, to evaluate the efficacy and safety of Qingpeng ointment for AGA. The primary objective is to evaluate the efficacy of Qingpeng ointment in relieving the joint pain and reducing the joint swelling of patients with AGA. The secondary objectives are to evaluate the efficacy of Qingpeng ointment in improving the joint mobility and relieving the joint redness in patients with AGA, and to evaluate the safety of Qingpeng ointment.

## Methods

### Study design

**Fig. 1:**
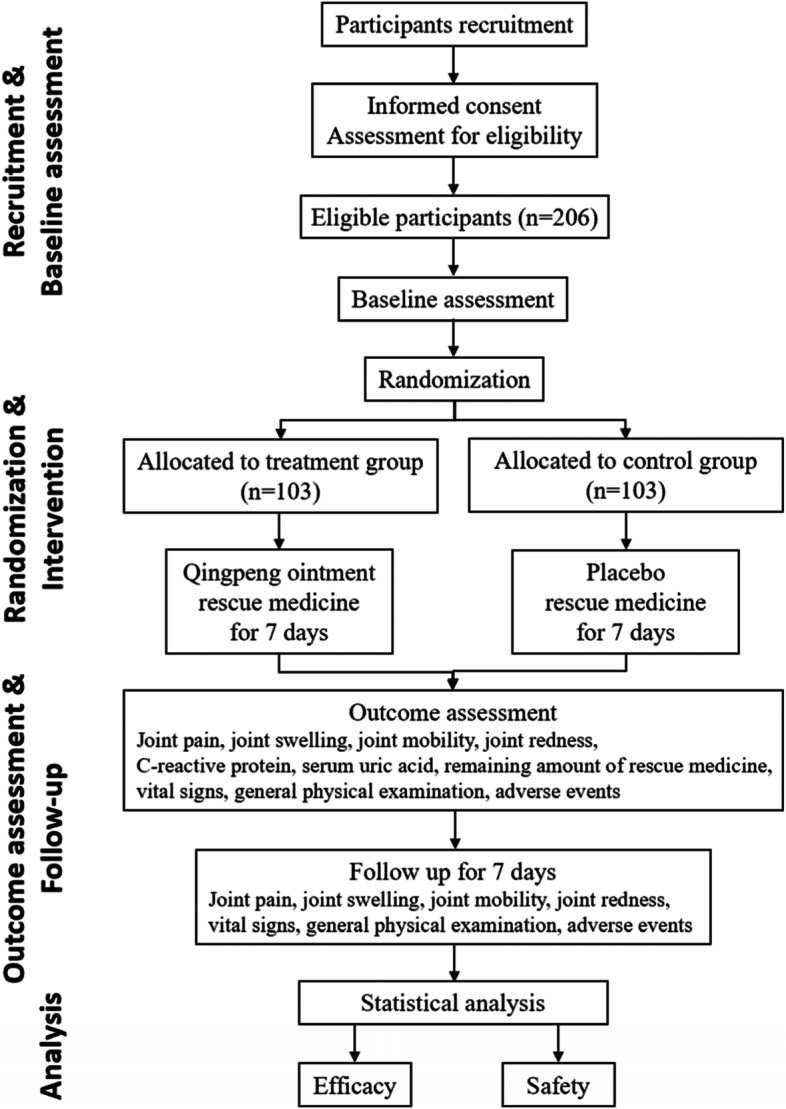
Trial flow chart

**Fig. 2:**
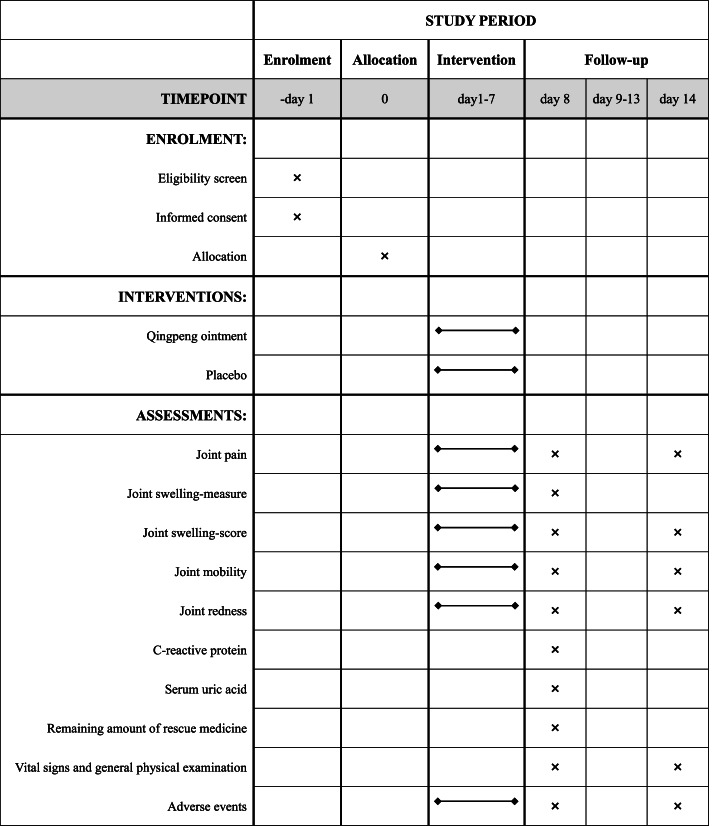
SPIRIT figure. Schedule of enrolment, interventions, and assessments

This study is a prospective, multi-center, randomized, double-blind, placebo-controlled trial. The study protocol was written in accordance with the Standard Protocol Items: Recommendations for Interventional Trials (SPIRIT) guidelines (Additional file 1 shows the SPIRIT checklist). The trial has been registered on ISRCTN registry: www.isrctn.com. The flowchart of the study design is shown in Fig. 1, and a detailed schedule of enrolment, intervention, and assessment is shown in Fig. 2.

### Study setting

This study will be conducted in three hospitals: Fangshan Hospital, Beijing University of Chinese Medicine in Beijing; the Second Affiliated Hospital of Zhejiang Chinese Medical University in Hangzhou, Zhejiang Province; and Liuzhou People’s Hospital in Liuzhou, Guangxi Zhuang Autonomous Region.

### Study process

#### Baseline visit

After signing an informed consent, patients will be interviewed to obtain information on demographic characteristics, history of past and present illness, and current medications. General physical examination (including height, weight, blood pressure, respiratory rate, heart rate, and temperature) and laboratory tests (including serum uric acid and C-reactive protein) will be conducted. Degree of joint pain, swelling, redness, and mobility will be scored, and width and thickness of affected joints will be measured. Patients who meet the eligibility criteria will be randomly assigned to treatment group and control group, with 103 patients in each group. Investigators will also instruct participants on how to score the degree of joint pain, swelling, redness and mobility, and how to measure the width and thickness of joints.

#### Treatment period

Patients in the treatment group will be treated with Qingpeng ointment, and patients in the control group will be treated with placebo ointment. Patients in both groups will be given diclofenac sodium sustained-release tablets (DSSRT) as rescue medicine. Drugs for other underlying diseases such as antihypertensive drugs, hypoglycemic drugs, and hypolipidemic drugs are permitted to be taken during the treatment period. And other Chinese patent medicine are prohibited during the trial. When participants experience severe skin allergies at application site, or experience serious adverse events, investigators will advise them to discontinue using the study drug. The degree of joint pain, swelling, redness, and mobility will be scored and recorded on patient diary every day by patients, and the width and thickness of affected joints will also be measured and recorded every day by themselves. The course of treatment is seven days.

#### Treatment visit

After 7 days of treatment, participants will return to hospital for treatment visit. General physical examination and laboratory tests (including serum uric acid, C-reactive protein) will be conducted. The degree of joint pain, swelling, redness, and mobility will be scored, and the width and thickness of affected joints will be measured. The combined medication and adverse events during treatment period will be recorded. The remaining study drugs will be recovered, drug distribution and recovery record form will be filled in, and the remaining amount of Qingpeng ointment/placebo ointment and rescue medicine will be recorded.

#### Follow-up visit

Seven days after treatment visit, participants will be followed up. The degree of joint pain, swelling, redness, and mobility will be scored, and the adverse events during this 7-day period will be recorded. The medications and treatment received during the follow-up period will also be recorded.

### Participants and recruitment

#### Recruitment and informed consent

Participants will be recruited from the outpatient department of the listed three hospitals. For all patients who come to the hospital for AGA and are likely to meet the eligibility criteria, investigators will introduce the entire study and informed consent to them, mainly including the study process and intervention methods. Patients will be informed that their participation in this study is entirely voluntary and they are free to withdraw from the study at any time. After signing informed consent and assessed for eligibility criteria, eligible patients will be invited to participate in the study.

#### Inclusion criteria

Patients will be eligible for this study if they fulfill the following inclusion criteria:

1. Meeting the ACR (American College of Rheumatology)/EULAR (European League Against Rheumatism) gout classification criteria [27]
2. Joint pain intensity score (visual analogue scale, VAS, 0–10 points) ≥3 points
3. With an acute flare of gout, the time from the onset to the visit to hospital should not exceed 1 week
4. Aged between 18 to 65 years old
5. Volunteer to participate in the study and sign the informed consent

#### Exclusion criteria

Patients will not be enrolled into this study if they meet the following exclusion criteria:

1. Also suffering from other types of arthritis
2. Allergic to the study drugs (Qingpeng Ointment, DSSRT)
3. With severe cardiovascular, cerebrovascular, liver, or kidney diseases
4. With mental diseases or senile dementia
5. Women during pregnancy and lactation
6. With skin ulceration at the affected joint(s)
7. Having newly added uric acid-lowering drugs for any reason in the past week
8. Participating in other clinical trials at the same time

#### Withdrawal or dropout criteria

Participants will withdraw from this study in the following cases:

1. Deciding to withdraw by themselves
2. No longer continuing to use the study drugs or accept visits
3. Having severe allergic reaction at the administration site
4. Experiencing serious adverse events or complications

### Interventions

#### Experimental group

Participants in treatment group will be treated with Qingpeng ointment (produced by Tibet Cheezheng Tibetan Medicine Co., Ltd; specification: 20g/ointment aluminum tube). The ingredients of Qingpeng ointment are given in Table 1. Every participant will be given three tubes of ointment. Participants should first clean the skin of affected joints and then apply the ointment to the skin. The dosage is that the ointment can cover the surface of affected joints, and the thickness is 0.3–0.5 cm. The applied part needs to be massaged gently until the ointment is totally absorbed by the skin. The duration of each application is approximately 0.5 h, the frequency of application is twice a day, and the interval between two applications is at least 8 h. The course of treatment is 7 days.

**Table 1:** Ingredients of Qingpeng ointment

Ingredient	Dose (g)
*Herba Oxytropis Falcatae* (Jidou)	100
*Rhei Spiciforme Randix* (Yadahuang)	50
*Radix Aconiti Flavi Et Penduli* (Tiebangchui)	75
*Chebulae Fructus* (Hezi)	100
*Terminaliae Belliricae Fructus* (Maohezi)	100
*Phyllanthi Fructus* (Yuganzi)	100
*Benzoinum* (Anxixiang)	35
*Caulis Tinosporae* (Kuanjinteng)	150
*Moschus* (Shexiang)	25

#### Placebo group

In order to evaluate the absolute efficacy and safety of Qingpeng ointment for AGA and reduce the risk of bias, we chose placebo as comparison. Participants in control group will be treated with placebo ointment (produced by Tibet Cheezheng Tibetan Medicine Co., Ltd). The ingredients of placebo ointment include purified water, liquid paraffin, glycerinum, methylparaben, and food coloring. The placebo ointment is the same as Qingpeng ointment in texture, color, smell, and packaging. The usage of placebo ointment is the same as Qingpeng ointment.

#### Rescue medicine

DSSRT (produced by Beijing Novartis Pharmaceutical Co., Ltd; specification: 75mg/tablet, 10 tablets/box) will be given to participants in both treatment group and control group as rescue medicine. Patients in both groups are recommended to take the rescue medicine only when the VAS score of joint pain is ≥ 7 points (scale of 0 to 10), which is scored by patients themselves. Patients will also be recommended not to take the rescue medicine when joint pain is reduced to a tolerable extent (VAS score reduce to less than 7 points). The recommended dose is one tablet per time, once daily, and the maximum dose is one tablet per time, twice a day.

### Assignment of interventions

#### Randomization

Participants will be randomly assigned to treatment group and control group at a ratio of 1:1, with 103 cases in each group. The random sequence was generated by an independent statistician, using the SAS 9.2 software.

#### Allocation concealment and blinding

Sequentially numbered drug containers of identical appearance were used to achieve allocation concealment. Two hundred and six drug boxes with identical appearance were numbered as 001–206. According to the random sequence, Qingpeng ointment and placebo ointment were put into the corresponding numbered boxes, with three tubes of Qingpeng ointment or placebo ointment in each box. One box of DSSRT was also put into each drug box at the same time. Special personnel are assigned to manage the study drugs in each hospital, and the corresponding sequence of drugs will be distributed to each patient according to their recruitment sequence. Throughout the study, the investigators and the participants will not know which group the participants are assigned to. For each participant sequence, a corresponding emergency letter was made, which records the allocation result of that participant. When a participant experience serious adverse events and the allocation result needs to be known, investigators can open the emergency letter. The process of unblinding, including reasons, time, and treatment results, should be recorded on the case report form.

### Outcomes

#### Primary outcomes

Because the objective of this study is to investigate the efficacy of Qingpeng ointment for relieving pain and swelling of joints in patients with AGA, changes in joint pain severity (measured using VAS), joint swelling degree (measured using VAS), and width and thickness of affected joints (measured using vernier calipers) between baseline and treatment completion will be considered as the primary outcomes.

1. Joint pain: Degree of joint pain will be measured by participants, using a visual analogue scale (VAS, 0–10 points, 0=no pain, 10=intolerable pain), at baseline, every day during treatment, and after 7-day treatment.
2. Joint swelling:
  2.1 The width and thickness of each affected joint will be measured using vernier calipers (brand: Ruineng, model: NR0139) at baseline, every day during treatment, and after 7-day treatment.
  2.2 Degree of joint swelling will be measured by participants, using a visual analogue scale (VAS, 0–10 points, 0=no swelling, 10=intolerable swelling), at baseline, every day during treatment, and after 7-day treatment.

#### Secondary outcomes

Secondary outcomes include changes in joint mobility degree and redness degree between baseline and treatment completion, changes in C-reactive protein and serum uric acid between baseline and treatment completion, and the remaining amount of rescue medicine at treatment completion.

1. Joint mobility: Degree of joint mobility will be measured by participants, using a 0–4 points scale (0=the mobility is normal, and is not restricted; 1=the mobility is slightly restricted, but normal activities can still be performed; 2=the mobility is moderately restricted, patient is unable to perform general activities, but is able to take care of self daily life; 3=the mobility is severely restricted, pain is unbearable when the joint moves, patient is unable to take care of self daily life; 4=the joint is unable to move), at baseline, every day during treatment, and after 7-day treatment.
2. Joint redness: Degree of joint redness will be measured by participants, using a 0-3 points scale (0=the skin color is normal; 1=the skin is slightly red; 2=the skin is obviously red; 3=the skin is dark red), at baseline, every day during treatment, and after 7-day treatment.
3. C-reactive protein: Will be measured from a blood sample taken at baseline and after 7 days of treatment.
4. Serum uric acid: Will be measured from a blood sample taken at baseline and after 7 days of treatment.
5. Remaining amount of rescue medicine: Will be recorded after 7 days of treatment.

#### Safety outcomes

1. Vital signs and general physical examination: Will be tested and recorded at baseline visit, treatment visit, and follow-up visit.
2. Adverse events: Adverse events during the trial will be recorded in detail.

### Sample size estimation

This study includes two primary outcomes: joint pain and joint swelling. Sample size was estimated based on these two primary outcome measures. Estimation according to joint pain was based on results from a previous study [23]. In this study, 47% participants in treatment group (Qingpeng ointment plus diclofenac sodium tablets) had significant relief of joint pain (the score of joint pain degree measured by a self-developed scale reduced by 6 points), and 25% participants in control group (diclofenac sodium tablets) had significant relief of joint pain. On the basis of a 5% type I error rate (*α*=0.05) and a power of 90% (*β*=0.1), the sample size of each group was 82. Assuming a 20% dropout rate, a total of 206 participants should be recruited, with 103 participants in each group.

Estimation according to joint swelling was likewise based on results from another previous study [19], which established score of joint swelling measured by a 0–3 points scale as 0.45 points after receiving Qingpeng ointment plus etoricoxib tablets, and the estimated standard deviation is 0.37. Score of joint swelling after receiving vaseline plus etoricoxib tablets is 0.87, and the estimated standard deviation is 0.64. On the basis of a 5% type I error rate (*α*=0.05) and a power of 90% (*β*=0.1), the sample size of each group was 27. Assuming a 20% dropout rate, a total of 68 participants should be recruited, with 34 participants in each group.

Based on these results, we chose the larger sample size of 206 participants, with 103 participants in each group.

### Data collection and management

Data will be collected on case report forms by investigators at baseline visit, treatment visit, and follow-up visit. During treatment period, data will be collected through patient diary. Reports of laboratory findings will also be collected and recorded. For participants who withdraw from the trial, the investigator will contact the participants as much as possible to obtain all measurable outcome data. Every investigator should collect and record the data carefully to guarantee its accuracy. Data will then be entered into EpiData manager by two investigators independently and checked to ensure the accuracy. Personal information of participants will be kept confidential. Every participant will be assigned a participant number, and all data will be identified using this number, instead of directly displaying personal information. All research documents will be saved for at least 5 years after the end of the trial.

### Statistical analysis

Statistical analysis will be performed using SAS 9.2 software, with a statistical significance of *p* value less than 0.05. Normally distributed continuous variables will be described using mean ± standard deviation (SD), and non-normally distributed continuous variables will be described using median and interquartile range (IQR). Categorical variables will be described using frequencies and percentages. Primary outcomes will be analyzed according to the intention-to-treat principle. Intra-group differences of continuous variables will be tested for significance using paired-samples *t* test when the data is normally distributed, and will be tested using non-parametric test when the data is non-normally distributed. Between-group differences of continuous variables will be tested for significance using independent-samples *t* test when the data of the two groups are normally distributed and have equal variance, and will be tested using non-parametric test when the data of the two groups are non-normally distributed or have unequal variances. Chi-square test will be used for categorical data. Data from participants who drop out from this study will be implemented by the last-observation-carried-forward (LOCF) method. Interim analyses will not be performed. For the co-primary endpoints, Bonferroni correction will be performed to correct multiplicity.

### Quality control

Before the start of the study, the investigators will be trained, so that they will have a consistent understanding of the study protocol and can teach participants how to score the degree of joint pain, swelling, redness, and mobility and how to measure the width and thickness of affected joints. The investigators will also be trained how to fill in the case report form, and they should fill in the form truthfully and completely as required. The original examination report or its copy during the study should be pasted on the case report form. Special personnel will be set up in each center, who will be responsible for the management, distribution, and recovery of the study drugs. The drug distribution and recovery record form will be filled in when distributing and recovering the study drugs. Center for Evidence-Based Chinese Medicine, Beijing University of Chinese Medicine, as the coordinating center of this trial, will assign clinical research associates to regularly conduct monitors at each study center, to monitor whether the investigators comply with the study protocol and fill in the case report forms as required, and whether the data collected is accurate.

### Ethics

This study will be completed in accordance with the ethical principles in the Declaration of Helsinki and the Good Clinical Practice (GCP) Guideline. Beijing University of Chinese Medicine is the sponsor of this trial and will monitor and audit this project. The study has been approved by ethics committee of Fangshan Hospital, Beijing University of Chinese Medicine, ethics committee of the Second Affiliated Hospital of Zhejiang Chinese Medical University, and ethics committee of Liuzhou People’s Hospital. If the protocol needs to be amended, main investigators of this study will meet together to discuss, communicate, and draw conclusions on the revision. The revised protocol will be submitted to the ethics committee of each study center for approval, and an application for revision will also be submitted to the trial registry.

### Dissemination policy

Results of this study will be published in a peer-reviewed journal. Authorship will be granted to authors who make important contributions to the creation of the final publication.

## Discussion

AGA is an inflammatory arthritis characterized by severe pain, swelling, and restricted movement of joints, with rising disease burden. The first-line treatment agents recommended in guidelines for AGA are colchicine, NSAIDs, and glucocorticoids, whose use may be limited by contraindications and adverse events [9–11]. In order to avoid adverse effects and to attain better efficacy, some complementary and alternative medicines are used to treat AGA. Qingpeng ointment is a Tibetan patent medicine for external use, which has the effects of promoting blood circulation, removing blood stasis, reducing swelling, and relieving pain, and can be used to relieve swelling and pain of joint and muscle caused by arthritis. Qingpeng ointment is convenient and safe to use, and only a few patients who are allergic to the drug may experience skin irritation such as itching, rash, and blister after using the drug. Results of several previous clinical trials indicated that Qingpeng ointment can relieve pain, swelling, redness, and dysfunction of joints in patients with AGA [18–23]. These studies were all small-sample, unblind, and based on one medical center, which may lead to bias caused by confounding factors. Only two studies reported using appropriate method for randomization [20, 23]. These studies mainly chose colchicine and NSAIDs (such as diclofenac sodium and etoricoxib) as the intervention for control group. There was no previous double-blind, placebo-controlled trial. In order to evaluate the efficacy and safety of Qingpeng ointment for patients with AGA more clearly, we designed this multi-center, randomized, double-blind, placebo-controlled trial.

In addition to Qingpeng ointment, there are some other ointments for promoting blood circulation, removing blood stasis, and relieving pain. These ointments are formed based on traditional Chinese medicine theory, some of which can be used to treat traumatic injuries, sprains and contusions, muscle soreness, joint swelling, and pain, such as Chuangshangning ointment and Jinluoning ointment, and some can be used to treat burns and scalds, such as Jingwanhong ointment. Similar to these ointments, Qingpeng ointment can also be used to treat sprains and contusions. The difference is that Qingpeng ointment can also relieve swelling and pain of joints and muscles caused by a variety of arthritis, such as rheumatic and rheumatoid arthritis, osteoarthritis, and gouty arthritis. It can also be used to treat eczema and skin itches. Secondly, different from those other ointments, Qingpeng ointment is a Tibetan medicine ointment, which is formed based on Tibetan medicine theory and using Tibetan medicine as raw material. Moreover, the efficacy of Qingpeng ointment for relieving swelling and pain caused by arthritis has been proved by several clinical studies. Among these studies, trials on AGA are few and have some methodological deficiencies. Therefore, in order to further clearly evaluate the efficacy and safety of Qingpeng ointment for AGA, we designed this study.

To the best of our knowledge, this study is the first multi-center, randomized, double-blind, and placebo-controlled clinical trial to assess the efficacy of Qingpeng ointment for AGA. In terms of outcome selection, considering from the perspective of patients, we chose joint pain and joint swelling as primary outcomes, and joint mobility, joint redness, remaining amount of rescue medicine, C-reactive protein, and serum uric acid as secondary outcomes. Different from previous studies, for the primary outcome of joint swelling, we will use vernier calipers to measure the width and thickness of affected joints, and evaluate the relief of joint swelling using the change of width and thickness. We will also use the visual analogue scale to measure the degree of joint swelling. Measuring joint swelling in these two ways is an innovation different from previous studies. Moreover, the intervention and control of this study are only Qingpeng ointment and placebo ointment. Therefore, in order to improve the compliance of participants, we chose DSSRT as a rescue medicine. Participants in both treatment group and control group can take rescue medicine when the VAS score of joint pain is ≥ 7 points, to relieve the pain. In this way, the compliance of participants can be improved, and the dropout rate of this study can be reduced. After the treatment period, the remaining amount of Qingpeng ointment/placebo ointment will be recorded to assess the compliance of participants to the treatment regimen, and the remaining amount of rescue medicine will also be used as a secondary outcome for evaluating the efficacy of Qingpeng ointment.

The limitation of this study is that the only intervention and control drug are Qingpeng ointment and placebo ointment. Patients with AGA may experience very severe joint pain. The compliance of participants may be affected, if only externally applied drugs are given. In order to improve the compliance of participants, DSSRT will be used as rescue medicine. The investigators will also explain the study protocol and medication requirements to participants detailedly, to ensure that the participants fully understand how to use the drugs. During the treatment period, participants will be instructed regularly to apply the drug on time, and come back to hospital for follow-up.

This study is expected to explicitly evaluate the efficacy and safety of Qingpeng ointment for AGA. If the results prove that Qingpeng ointment can effectively relieve symptoms of AGA, and is safe to use, then the application of Qingpeng ointment in the treatment of AGA can be promoted.

## Trial status

This is protocol version 3.0, dated August 2020. The study is currently actively recruiting eligible participants. Recruitment started on March 2021 and is expected to be completed on December 2021.

## Supplementary Information

**Additional file 1.** SPIRIT 2013 Checklist: Recommended items to address in a clinical trial protocol and related documents.

## Data Availability

The data-sharing plans for this study are unknown and will be made available based on the contract with the company.

## Abbreviations

AGA: Acute gouty arthritis
VAS: Visual analogue scale
NSAID: Nonsteroidal anti-inflammatory drug
TTM: Traditional Tibetan medicine
DSSRT: Diclofenac sodium sustained-release tablets
SD: Standard deviation
IQR: Interquartile range
LOCF: Last-observation-carried-forward
GCP: Good Clinical Practice
